# H_3_P···AgI: generation by laser-ablation and characterization by rotational spectroscopy and *ab initio* calculations†
†Electronic supplementary information (ESI) available: All underlying data are provided as electronic supplementary information accompanying this paper. See DOI: 10.1039/c6cp03512d



**DOI:** 10.1039/c6cp03512d

**Published:** 2016-06-20

**Authors:** Susanna L. Stephens, David P. Tew, Nicholas R. Walker, Anthony C. Legon

**Affiliations:** a School of Chemistry , Newcastle University , Bedson Building , Newcastle-upon-Tyne , NE1 7RU , UK . Email: nick.walker@newcastle.ac.uk; b School of Chemistry , University of Bristol , Cantock's Close , Bristol , BS8 1TS , UK . Email: a.c.legon@bristol.ac.uk

## Abstract

The new compound H_3_P···AgI has been synthesized in the gas phase by means of the reaction of laser-ablated silver metal with a pulse of gas consisting of a dilute mixture of ICF_3_ and PH_3_ in argon. Ground-state rotational spectra were detected and assigned for the two isotopologues H_3_P···^107^AgI and H_3_P···^109^AgI in their natural abundance by means of a chirped-pulse, Fourier-transform, microwave spectrometer. Both isotopologues exhibit rotational spectra of the symmetric-top type, analysis of which led to accurate values of the rotational constant *B*_0_, the quartic centrifugal distortion constants *D*_*J*_ and *D*_*JK*_, and the iodine nuclear quadrupole coupling constant *χ*_*aa*_(I) = *eQq*_*aa*_. *Ab initio* calculations at the explicitly-correlated level of theory CCSD(T)(F12*)/aug-cc-pVDZ confirmed that the atoms P···Ag–I lie on the *C*_3_ axis in that order. The experimental rotational constants were interpreted to give the bond lengths *r*_0_(P···Ag) = 2.3488(20) Å and *r*_0_(Ag–I) = 2.5483(1) Å, in good agreement with the equilibrium lengths of 2.3387 Å and 2.5537 Å, respectively, obtained in the *ab initio* calculations. Measures of the strength of the interaction of PH_3_ and AgI (the dissociation energy *D*_e_ for the process H_3_P···AgI = H_3_P + AgI and the intermolecular stretching force constant *F*_P···Ag_) are presented and are interpreted to show that the order of binding strength is H_3_P···HI < H_3_P···ICl < H_3_P···AgI for these metal-bonded molecules and their halogen-bonded and hydrogen-bonded analogues.

## Abstract 2

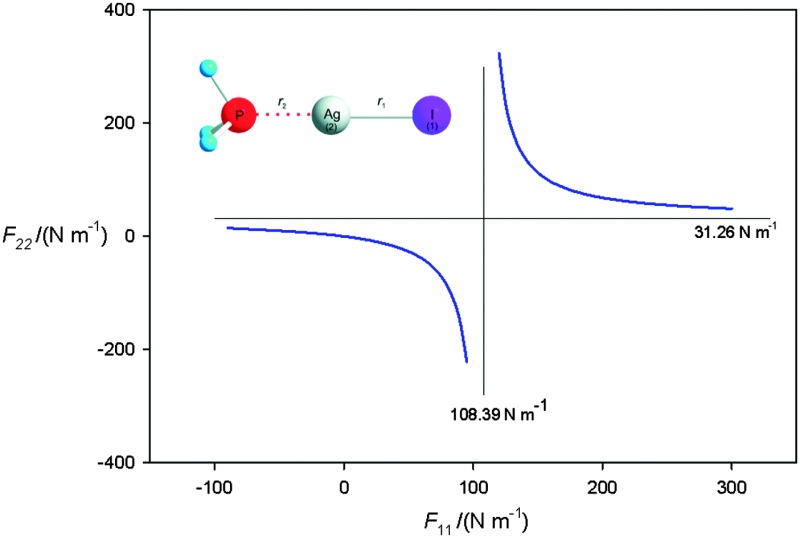
The new compound H_3_P···AgI has been synthesized in the gas phase by means of the reaction of laser-ablated silver metal with a pulse of gas consisting of a dilute mixture of ICF_3_ and PH_3_ in argon.

## 1 Introduction

A programme of systematic investigations of small molecules of the type B···MX is being conducted, where B is a small Lewis base (*e.g.* N_2_, OC, H_2_O, H_2_S, HC≡CH, H_2_C=CH_2_, cyclopropane or NH_3_), M = Cu, Ag or Au, and X = F, Cl or I.^1–19^ The programme has both experimental and theoretical components. The experimental approach is to produce B···MX by laser ablation of the metal M in the presence of a gas pulse composed of small amounts of B and a molecular source of halogen atoms X in a large excess of argon. Following supersonic expansion of the product B···MX entrained in the carrier gas, its rotational spectrum is observed in isolation at a low effective temperature. Various properties of B···MX are available through analysis of the rotational spectrum, namely the angular geometry, the distances *r*(B···M) and *r*(M–X), the strength of the intermolecular bond B···M, and the electric charge redistribution that accompanies formation of B···MX. The theoretical component of the investigations involves *ab initio* calculations at the CCSD(T)(F12*) explicitly correlated level of theory, usually with the largest basis set affordable. These calculations have the advantage of providing accurate properties of the isolated molecule, which can be compared with the experimental results.

Several molecules H_3_N···MX, where M = Cu or Ag and X = F, Cl or I, have been detected and characterised recently in the gas phase for the first time through their rotational spectra,^17–19^ although H_3_N···CuCl was identified in the solid state earlier.^20^ Each was established to be a symmetric-top molecule, with the N···MX nuclei lying on the top (*C*_3_) axis, in the order indicated. To date, analogues of H_3_N···MX having phosphine instead of ammonia as the Lewis base B have not been identified experimentally, to the best of our knowledge, but several have been the subject of density functional calculations.^21,22^ We report here the rotational spectrum of H_3_P···Ag–I and some of its properties derived therefrom.

There is some evidence that molecules B···MX (M = Cu, Ag, or Au; X = F, Cl, or I)^1–19^ have geometries that are isomorphic with those of their hydrogen-bonded (B···HX, X is a halogen atom)^23^ and halogen-bonded (B···XY, XY is a dihalogen molecule)^24^ counterparts, but are more strongly bound and exhibit a greater electric charge rearrangement within the diatomic subunit. Our interest here is to examine the geometry and binding strength of H_3_P···Ag–I and the electric charge redistribution within Ag–I that accompanies its formation. These properties will then be compared with those of the closely related molecule H_3_N···CuI,^19^ with those of their hydrogen-bonded analogues H_3_P···HI^25^ and H_3_N···HI^26^ and with those of their halogen-bonded relatives, H_3_P···ICl^27^ and H_3_N···ICl.^28^

## 2 Experimental and theoretical methods

### 2.1 Detection of the rotational spectrum

A chirped-pulse Fourier-transform microwave (CP-FTMW) spectrometer fitted with a laser ablation source was used to observe rotational spectra in the frequency range 6.5 to 18.5 GHz. Detailed descriptions of the spectrometer and laser ablation source are available elsewhere.^29,30^ A gas sample containing ∼4.0% PH_3_ and ∼1.5% CF_3_I in argon was prepared at a total pressure of 6 bar. The sample was pulsed over the surface of a silver rod that was ablated by a suitably timed Nd:YAG laser pulse (wavelength 532 nm, pulse duration 10 ns, pulse energy 20 mJ). Subsequently, the gas pulse expanded supersonically into the vacuum chamber of the spectrometer. The rod was translated and rotated regularly at small intervals to allow each laser pulse (repetition rate of ∼1.05 Hz) to impinge on a fresh metal surface and thereby ensure shot-to-shot reproducibility.

The sequence employed to record broadband microwave spectra involves repetition of two steps. The first is polarization of the sample by a microwave chirp that sweeps from 6.5 to 18.5 GHz within 1 μs and the second is recording of the subsequent free induction decay of the molecular emission over a 20 μs time period. This sequence is repeated eight times during the expansion of each gas sample pulse into the spectrometer chamber. The free induction decay (FID) of the polarization is mixed down with the signal from a 19 GHz local oscillator and then digitized by means of a 25 Gs s^–1^ digital oscilloscope. Each transition is observed as a single peak with full-width at half-maximum (fwhm) ≅ 150 kHz after application of a Kaiser–Bessel digital filter.

### 2.2 *Ab initio* calculations

Structure optimizations and counter-poise corrected dissociation energies were calculated using the Turbomole package^31^ at the CCSD(T)(F12*) level of theory,^32^ a coupled-cluster method with single and double excitations, explicit correlation,^33^ and a perturbative treatment of triple excitations.^34^ Only valence electrons were included in the correlation treatment. A basis set combination consisting of aug-cc-pVDZ on H and P atoms and aug-cc-pVDZ-PP on Ag and I atoms was used and will be referred to by AVDZ. ECP-10-MDF^35,36^ and ECP-28-MDF^37^ were used on Ag and I, respectively, to account for scalar relativistic effects. For the density fitting approximation used to accelerate the CCSD(T)(F12*) calculation, the respective def2-QZVPP basis sets were employed for the MP2^38,39^ and Fock^40^ terms. For the complementary auxiliary basis required for the F12 treatment,^41^ the aug-cc-pCVDZ MP2 density fitting basis sets were used.^39^ Quadratic force constants were also calculated at this level of theory. For comparison, the same force constants were calculated with the GAUSSIAN 09 package^42^ at the MP2 level of theory. A basis set combination consisting of aug-cc-pVTZ on the H and P atoms, and aug-cc-pVTZ-PP on the Ag and I atoms was used in this case.

## 3 Results

### 3.1 Determination of spectroscopic constants

**Fig. 1:**
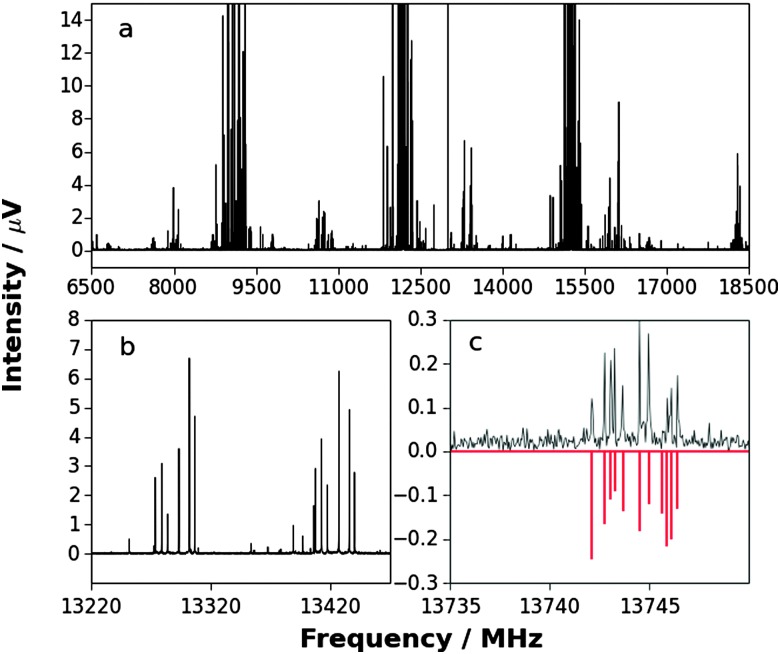
Top panel: (a) broadband spectrum recorded while probing a sample containing CF_3_I, Ag and PH_3_ (530k FIDs). Some transitions of CF_3_I are taken off-scale to allow weaker transitions to be distinguished from the baseline. (b) Expanded section of spectrum displayed in (a) to show *J*′ – *J*′′ = 5–4 transitions of ^107^AgI (∼13 420 MHz) and ^109^AgI (∼13 280 MHz). Hyperfine splittings in each transition are evident. (c) Expanded section of the spectrum displayed in (a) to show *J*′ – *J*′′ = 11–10 transition of H_3_P···^109^AgI (black). A simulated spectrum that uses the results of fitted parameters is displayed inverted (red).

The observed spectrum of H_3_P···AgI showed evidence of the presence of the two isotopologues H_3_P···^107^AgI and H_3_P···^109^AgI, each exhibiting iodine nuclear quadrupole hyperfine structure, as may be seen from consideration of Fig. 1. An iterative least-squares fit of the observed hyperfine frequencies of each isotopologue was conducted using the program PGOPHER, written and maintained by Western.^43^ The Hamiltonian employed was of the form
1

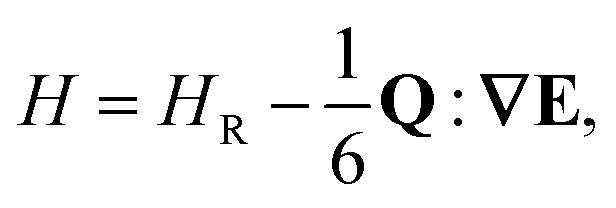

where *H*_R_ is the usual energy operator appropriate to a semi-rigid symmetric rotor molecule and –⅙**Q**:**∇E** is the iodine nuclear quadrupole energy operator, in which **Q** is the iodine nuclear electric quadrupole moment tensor and **∇E** is the electric field gradient tensor at I. The matrix of *H* was constructed in the coupled symmetric-rotor basis ***I*** + ***J*** = ***F***. The only determinable spectroscopic constants were the rotational constant *B*_0_, the quartic centrifugal distortion constants *D*_*J*_ and *D*_*JK*_, and the iodine nuclear quadrupole coupling constant *χ*_*aa*_(I) = –*eQ*∂^2^ *V*/∂*a* ^2^ = *eQq*_*aa*_ (where *q*_*aa*_ = –∂^2^ *V*/∂*a* ^2^ is the electric field gradient along the *C* *a*3 axis direction). The magnetic coupling of the iodine nuclear spin to the molecular rotation can in principle be described by the spin-rotation constant *C*_*bb*_ but this constant was too small to determine from the observed frequencies. Values of the spectroscopic constants from the final cycle of the least-squares fit with PGOPHER are given in Table 1 for the two isotopologues H_3_P···^107^AgI and H_3_P···^109^AgI investigated, together with *σ*_RMS_, the RMS deviation of the fit, and *N*, the number of hyperfine components fitted. Spectra simulated using PGOPHER and the final set of spectroscopic constants are shown in Fig. 1. The detailed PGOPHER fits are available as Supplementary Material. The values of *σ*_RMS_ are satisfactory, given the estimated accuracy of frequency measurement (12 kHz) associated with the chirped-pulse F-T microwave spectrometer.

**Table 1:** Observed spectroscopic constants^*a*^ of H_3_P···^107^AgI and H_3_P···^109^AgI

Spectroscopic constant	H_3_P···^107^AgI	H_3_P···^109^AgI
*B*_0_/MHz	626.01307(23)	624.76423(17)
*D*_*J*_/kHz	0.03182(89)	0.03238(64)
*D*_*JK*_/kHz	4.46(14)	4.04(10)
*χ*_*aa*_(I)/MHz	–733.83(34)	–734.54(27)
*σ*_RMS_ ^*b*^/kHz	12.0	9.0
*N* ^*c*^	88	93

^*a*^Numbers in parentheses are one standard deviation in units of the last significant digits.

^*b*^Standard deviation of the fit.

^*c*^Number of hyperfine frequencies included in the fit.

### 3.2 Molecular geometry

**Fig. 2:**
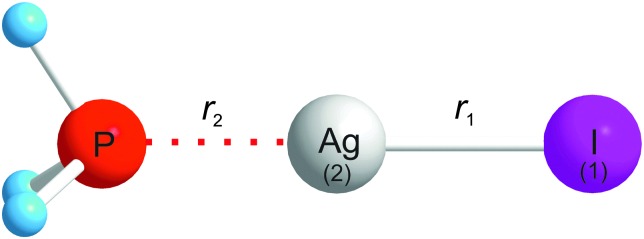
The molecular geometry of H_3_P···AgI drawn to scale. The internal coordinates *r*_1_ and *r*_2_ used in the discussion of how to obtain force constant *F*_22_ from the centrifugal distortion constants *D*0J are indicated. The experimental zero-point values of *r*_1_ and *r*_2_ are *r*_0_(Ag–I) = 2.5483(1) Å and *r*_0_(P···Ag) = 2.3488(20) Å, respectively.

The facts that the ground-state rotational spectrum of the detected complex of phosphine and argentous iodide is of the symmetric-top type and that the Ag atom is close to the complex centre of mass (see later) mean that the arrangement of the atoms is either H_3_P···AgI or PH_3_···AgI. The second of these is unlikely because ^*δ*+^Ag–I^*δ*–^ is dipolar in the indicated sense and it is expected that the positive end of the electric dipole would interact with the P non-bonding electron pair, which lies on the *C*_3_ axis of phosphine. This expectation is confirmed by *ab initio* calculations at the CCSD(T)(F12*)/AVDZ level of theory, which predict that the optimised geometry of PH_3_···AgI lies higher in energy by 116 kJ mol^–1^ than that of the H_3_P···AgI conformer. The higher energy conformer would not be populated at the low effective temperature (∼2 K) of the supersonic expansion. The observed conformer is therefore of the general form shown in Fig. 2.

The rotational constants *B*_0_ = *C*_0_ for the two isotopologues H_3_^31^P···^107^Ag^127^I and H_3_^31^P···^109^Ag^127^I allow only a partial determination of the lengths of the H–P, P···Ag and Ag–I bonds and of the angle *α* = ∠HPAg (between the P–H bond and the *C*_3_ axis) necessary to define the *r*_0_ geometry. The quantities of most interest are *r*_0_(P···Ag) and *r*_0_(Ag–I). The *ab initio* calculations indicate that *r*_e_(P–H) decreases by 0.0114 Å when phosphine enters the complex and the angle *α*_e_ decreases by 3.94°. We shall assume that the *r*_0_ geometry of phosphine (*r*_0_(P–H) = 1.420003 Å and angle *α*_0_ = 122.86° obtained by fitting the accurately known^44^ *B*_0_ and *C*_0_ using the STRFIT program of Kisiel^45^) changes in the same way as does the *r*_e_ geometry on formation of H_3_P···Ag–I. If so, *r*_0_(P–H) = 1.4086 Å and *α*_0_ = 118.92° are appropriate to PH_3_ in the complex. When these values were assumed in a fit of the ground-state principal moments of inertia of H_3_^31^P···^107^Ag^127^I and H_3_^31^P···^109^Ag^127^I, the values *r*_0_(P···Ag) = 2.3488 Å and *r*_0_(Ag–I) = 2.5483 Å resulted. No errors in these quantities are generated in the fit because two constants are fitted by two parameters. However, calculations reveal the following variations: ∂*r*(P···Ag)/∂*r*(P–H) = 0.065, ∂*r*(Ag–I)/∂*r*(P–H) = 0.005, ∂*r*(P···Ag)/∂*α* = 0.002 Å deg^–1^. and ∂*r*(Ag–I)/∂*α* = 0.0001 Å deg^–1^. Thus, the length *r*_0_(Ag–I) is very insensitive to changes to the geometry of PH_3_ that might occur when H_3_P···AgI is formed. These partial derivatives lead, when the reasonable errors of δ*r*_0_ = 0.005 Å and δ*α*_0_ = 1° are assumed, to *r*_0_(P···Ag) = 2.3488(20) Å and *r*_0_(Ag–I) = 2.5483(1) Å. The results from the CCSD(T)(F12*)/AVDZ optimisation of H_3_P···AgI are 2.3387 Å and 2.5537 Å, respectively.

The fact that spectroscopic constants have been determined for the isotopologues H_3_P···^107^AgI and H_3_P···^109^AgI allows the coordinate *a*_Ag_ to be obtained by the substitution method from the expression

2 *a*_Ag_^2^ = Δ*I*0*b*/*μ*_s_,

in which Δ*I*0*b* is the difference in the zero-point moments of inertia of the two isotopologues and 
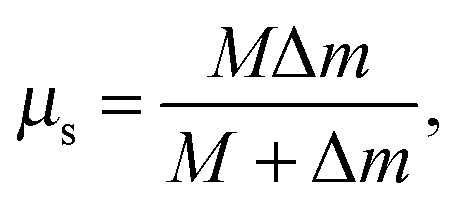
 where *M* is the mass of the parent and Δ*m* is the mass change accompanying the isotopic substitution at Ag. The result is |*a*_Ag_| = 0.9017(17) Å, where the error is estimated from δ*a* = 0.0015/|*a*| as recommended by Costain.^46,47^ The corresponding values for this coordinate implied by the determined *r*_0_ geometry and the *ab initio r*_e_ geometry are 0.9017 Å and 0.9056 Å, respectively.

### 3.3 Strength of the interaction of H_3_P and AgI

There are two common measures of the strength of the interaction of phosphine and silver iodide in H_3_P···AgI. Both are properties of the one-dimensional potential-energy function associated with variation of the distance *r*(P···Ag) when *C*_3v_ symmetry is maintained but with structural relaxation at each point (referred to as the dissociation coordinate). The first is the intermolecular stretching quadratic force constant *F*_P···Ag_. The second is the energy, *D*_e_, required to dissociate H_3_P···AgI to give PH_3_ and AgI at infinite separation, with reactants and products at their equilibrium geometries. The first can be obtained from the experimental centrifugal distortion constants *D*_*J*_ but the second is not available from the present experiments. Both are available from the *ab initio* calculations.

For weakly bound complexes (such as most hydrogen-bonded complexes B···HX, where B is a simple Lewis base and X is a halogen atom) it is a good approximation to assume that B and HX are rigid and unchanged in geometry on complex formation. Then *F*_B···H_ can be related to the equilibrium centrifugal distortion constant *D*e*J* or *Δ*e*J* (depending on molecular symmetry) of the complex and the various rotational constants of B, HX and B···HX, as demonstrated by Novick^48^ for the case where B is an atom and by Millen^49^ for a wider range of molecules B. For complexes B···MX, where M is a coinage metal atom, the intermolecular bond can be strong and the approximation that the force constant *F*_B···M_ is much smaller than all other stretching force constants is no longer appropriate. To deal with such cases, we have recently described a two-force constant model which relates the quadratic force constants *F*_M–X_ and *F*_B···M_ (hereafter referred to as *F*_11_ and *F*_22_, respectively) to either *D*e*J* or *Δ*e*J* under the assumption that the contribution of the cross term *F*_12_ is negligible.^50^ The model applies to all complexes of a Lewis base B with any diatomic molecule (*e.g.* a hydrogen halide HX, a dihalogen XY, or a coinage metal halide MX) as long as the diatomic molecule lies along a *C*_*n*_ (*n* ≥ 2) symmetry axis of B in the equilibrium geometry. Note that B is assumed rigid, but can be changed in geometry when subsumed into the complex. During the vibrational motion no further change is assumed, however.

The two-force constant model for a symmetric-top molecule such as H_3_P···AgI leads (with numbering of the Ag and I atoms and internal coordinates *r*_1_ and *r*_2_ shown in Fig. 2) to the expression^50^

3

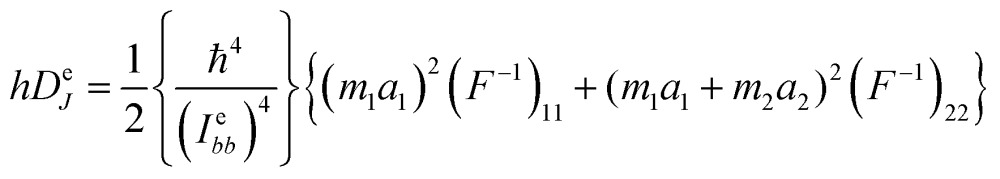

**Fig. 3:**
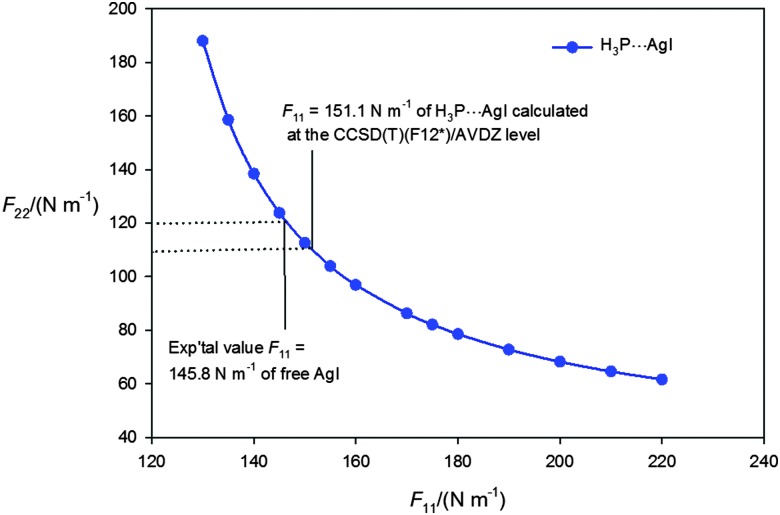
Values of the quadratic intermolecular stretching force constant *F*_22_ obtained by fitting the centrifugal distortion constants *D*0J of the isotopologues H_3_P···^107^AgI and H_3_P···^109^AgI using eqn (3) at fixed values of the AgI stretching force constant *F*_11_ in the range 130 to 220 N m^–1^. Eqn (3) is valid only if the off-diagonal force constant *F*_12_ is assumed to be zero. The CCSD(T)(F12*)/AVDZ-F12 value of *F*_11_, the experimental equilibrium value for the free AgI molecule and the *F*_22_ values they correspond to are indicated.

In eqn (3), *I*e*bb* is an equilibrium principal moment of inertia and the *a*_*n*_ are equilibrium principal axis coordinates of atoms *n* = 1 and 2. The compliance matrix elements (*F* ^–1^)_*nn*_ are simply 1/*F*_*nn*_ under the approximations described above. It was shown in ref. 50 that zero-point constants and coordinates can be used in place of equilibrium values to a reasonable approximation. Least-squares fitting of (*F* ^–1^)_11_ and (*F* ^–1^)_22_ simultaneously to the *D*0*J* values of the two isotopologues H_3_P···^107^AgI and H_3_P···^109^AgI led to ill-conditioning, however, so instead a fixed value of *F*_11_ was assumed and *F*_22_ was fitted. Fig. 3 shows *F*_22_ plotted as a function of *F*_11_ for a wide range of values of the latter, with the equilibrium value of the force constant 145.8 N m^–1^ of the free diatomic molecule Ag–I indicated, as calculated from its equilibrium vibrational wavenumber.^51^ If it is assumed that *F*_11_ is unchanged from the equilibrium value in free AgI of 145.8 N m^–1^, the result is *F*_22_ = 122(5) N m^–1^, where the error is that transmitted from the fit of the *D*0*J* values.

It is also possible to calculate *F*_11_ and *F*_22_ *ab initio*. At the CCSD(T)(F12*)/AVDZ level of theory the results are *F*_11_ = 151.1 N m^–1^ and *F*_22_ = 106.8 N m^–1^. When the *D*0*J* values are fitted by using eqn (3) with *F*_11_ fixed at 151.1 N m^–1^, the result is *F*_22_ = 110(5) N m^–1^, where the error is that implied by the error in the *D*0*J* values, and is the best present experimental estimate for this quantity. For free AgI at the same level of theory, *F*_11_ = 145.9 N m^–1^ is obtained, in excellent agreement with the experimental equilibrium value of 145.8 N m^–1^. Thus. *F*_11_ increases by 3.5% when AgI is incorporated into H_3_P···AgI. For comparison, the lower level of theory MP2/aug-cc-pVTZ-PP gives *F*_11_ = 168.8 N m^–1^ and *F*_22_ = 130.1 N m^–1^ when using the GAUSSIAN package.^42^ The result for free AgI at the same level is *F*_11_ = 160.1 N m^–1^, corresponding to 9.8% overestimation of the experimental equilibrium value. If *F*_11_ for H_3_P···AgI were also overestimated by a similar percentage, the corrected value would be *F*_11_ = 153 N m^–1^, which likewise represents a small increase relative to that of the free molecule.

It has been shown that in the limit of rigid, unchanged B and MX geometries, when *F*_11_ becomes infinite, eqn (3) reduces to the corresponding Millen expression^49^

4

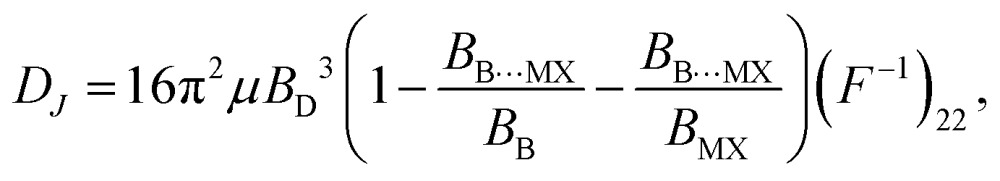

in which *B*_B···MX_, *B*_B_ and *B*_MX_ are equilibrium rotational constants of the complex and its components, but zero-point values are used of necessity. In eqn (4), *μ* = *m*_B_ *m*_MX_/(*m*_B_ + *m*_MX_).

**Fig. 4:**
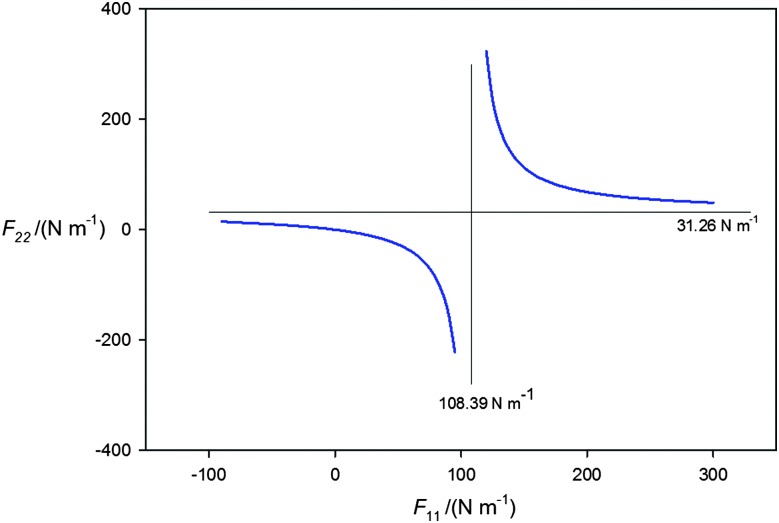
The rectangular hyperbola obtained by following the procedure described in the caption to Fig. 3, but with the range of assumed *F*_11_ values extended from –100 to +300 N m^–1^. The negative values of *F*_22_ and *F*_11_ are unphysical. The asymptote at *F*_11_ = 108.39 N m^–1^ represents the value of that force constant below which a negative, unphysical value of *F*_22_ is required to fit the centrifugal distortion constants *D*0*J*. The asymptote at *F*_22_ = 31.26 N m^–1^ is the value of this force constant in the limit *F*_11_ = ∞ N m^–1^, that is when the MX molecule is rigid. It can be shown^50^ that if both PH_3_ and AgI were rigid and unperturbed on formation of H_3_P···AgI eqn (3) leads to the Millen eqn (4), when equilibrium spectroscopic constants are used in the latter.

When B = H_3_P and MX = AgI, a fit of the centrifugal distortion constants *D*0*J* of H_3_P···^107^AgI and H_3_P ^109^AgI (using zero-point rotational constants given in Tables 1 and 2) leads to *F*_22_ = 31.3(5) N m^–1^, which is a very serious underestimate. The reason why becomes clear when the plot of *F*_22_ as a function of *F*_11_ is extended to cover a wider range of *F*_11_ values and unphysical solutions for which *F*_22_ is negative are included. The result is the rectangular hyperbola shown in Fig. 4. The horizontal asymptote (*F*_11_ = ∞) gives *F*_22_ = 31.26 N m^–1^ and corresponds to the solution when AgI is rigid and unperturbed when within H_3_P···AgI. The vertical asymptote (108.39 N m^–1^) corresponds to the lowest possible value of *F*_11_ consistent with the observed *D*0*J*. Clearly, any reasonable *F*_11_ must lead to a *F*_22_ value that is considerably greater than that given by eqn (4).

**Table 2:** Some properties of H_3_P and Ag–I

Property	H_3_P^*a*^	Property	^107^AgI^*b*^	^109^AgI^*b*^
*B*_0_/MHz	133480.1165(17)	*B*_0_/MHz	1342.99237(7)	1329.61831(7)
*C*_0_/MHz	117489.4357(77)	*χ*_*aa*_(I)/MHz	–1062.5299(15)	–1062.5230(14)
*r*_0_(P–H)/Å	1.42000^*c*^	*r*_0_(Ag–I)/Å	2.546627	2.546617
∠(HPH)/°	93.345^*c*^	*F*_AgI_/(N m^–1^)	145.78(3)^*d*^	145.76(3)^*d*^

^*a*^ Ref. 44.

^*b*^ Ref. 55.

^*c*^Calculated by fitting the zero-point rotational constants using the program STRFIT (ref. 45).

^*d*^Calculated from the equilibrium vibrational wavenumber *ω*_e_ given in ref. 51 by using the expression *F*_AgI_ = 4π^2^ *ω*_e_^2^ *c* ^2^ *μ*_AgI_, where *μ*_AgI_ = *m*_Ag_ *m*_I_/(*m*_Ag_ + *m*_I_).

The other measure of the strength of binding is the dissociation energy defined earlier; it takes the value *D*_e_ = 116 kJ mol^–1^ when calculated at the CCSD(T)(F12*)/AVDZ level of theory, after counterpoise correction.^52^ The value for AgI = Ag + I at the same level of theory is 230 kJ mol^–1^. It is therefore clear from the *D*_e_ value and the force constant *F*_22_ that the intermolecular bond in H_3_P···AgI is by no means weak. In fact by either measure, the P···Ag bond is about an order of magnitude stronger than most hydrogen or halogen bonds, but is only about a factor of two weaker than the Ag–I bond itself.

### 3.4 Electric charge redistribution on formation of H_3_P···AgI

The iodine nuclear quadrupole coupling constant *χ*_*aa*_(I) = *eq*I*aa* *Q* ^I^ carries information about the electric charge distribution at I through the electric field gradient *q*I*aa* along the *a*-axis direction at the iodine nucleus. According to the Townes–Dailey model^53^ for interpreting such coupling constants, the ionicity *i*_c_ (or fractional ionic character) of the free AgI molecule is given by
5

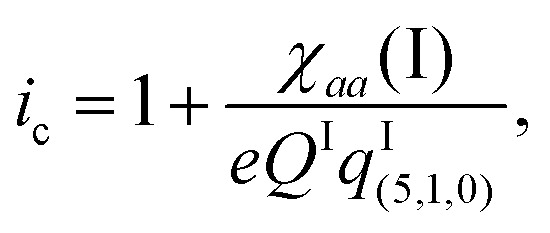

in which *q*I(5,1,0) is the contribution to the electric field gradient at I along the *a*-axis direction that arises from an electron in 5p_*a*_ orbital. The quantity *eQ* ^I^ *q*I(5,1,0) has the value 2292.71 MHz when described as a frequency.^54^Eqn (5) leads to the result *i*_c_ = 0.537 for ^107^Ag^127^I (the values^51,55^ of several properties of AgI, including *χ*_*aa*_(I), are collected in Table 2) but has the value 0.680 for the complex H_3_P···^107^Ag^127^I. Evidently, the charge rearrangement within AgI on formation of the complex is significant, a result consistent with the similar magnitude of the values for the dissociation energies *D*_e_ for the processes H_3_P···AgI = H_3_P + AgI and AgI = Ag + I referred to earlier. Interestingly, there appears to be very little change in its bond length when AgI is subsumed into the complex.

## 4 Conclusions

The new molecule H_3_P···Ag–I has been synthesized in the gas phase by a laser ablation method in which a pulse of gas mixture consisting of a few per cent each of PH_3_ and ICF_3_, with the remainder Ar, interacts with the plasma produced when silver is ablated by a Nd-YAG laser operating at 532 nm. The product was detected and characterised by means of its rotational spectrum, as observed with a chirped-pulse, Fourier-transform microwave spectrometer. The molecule is a symmetric top of *C*_3v_ symmetry with the atoms P, Ag and I lying in that order on the symmetry axis *a*. Spectroscopic constants determined by fitting the observed transitions of the isotopologues H_3_P···^107^AgI and H_3_P···^109^AgI were interpreted to give the values *r*_0_(P···Ag) = 2.3488(20) Å and *r*_0_(Ag–I) = 2.5483(1) Å for the indicated bond lengths, after assuming changes to the *r*_0_ geometry of free PH_3_ when bound up in the complex were the same as the corresponding changes in the *r*_e_ geometry, as predicted *ab initio* at the CCSD(T)(F12*)/AVDZ level of theory. It is of interest to note that the value of *r*_0_(Ag–I) is increased by only 0.0017 Å relative to the free AgI value of 2.54663 Å (see Table 2).^55^ Interpretation of the centrifugal distortion constants *D*0*J* and the iodine nuclear quadrupole coupling constants led to a value *F*_22_ = 110 (5) N m^–1^ for the quadratic stretching force constant of the P···Ag bond and to a value δ*i*_c_ = 0.14 for the increase in the Ag–I bond ionicity when H_3_P···AgI is formed. Although the ionicity of AgI increases significantly when subsumed into the complex, we note that the length of the bond and its force constant *F*_11_ are effectively unchanged.

H_3_N···CuI, synthesized and characterised recently by a similar method,^19^ is isomorphic with H_3_P···AgI and has *r*_0_(N···Cu) = 1.9357(13) Å and *r*_0_(Cu–I) = 2.3553(5) Å, the latter representing an increase of only 0.0147 Å relative to the free Cu–I value of 2.34059 Å. The N···Cu interaction strength, as measured by *F*_22_ = 110(30) N m^–1^, is similar to that 110(5) N m^–1^ of P···Ag in H_3_P···AgI, but the *ab initio* value for the other measure of binding strength for H_3_N···CuI (*D*_e_ = 168 kJ mol^–1^) is significantly larger than that (116 kJ mol^–1^) of H_3_P···AgI. The increase, δ*i*_c_ = 0.14, in the ionicity of the Cu–I bond when H_3_N···CuI is formed is identical to that observed for H_3_P···AgI. We conclude that H_3_P···AgI and H_3_N···CuI are very similar in their properties: both are strongly bound, both have similar changes in the ionicity of the M–I bond when the free MI molecule is subsumed into the complex, but the bond length *r*_0_(M–I) is effectively unchanged in both by this process.

Several complexes involving hydrogen bonds and halogen bonds to ammonia and phosphine have been described elsewhere, namely H_3_P···HI,^25^ H_3_N···HI,^26^ H_3_P···ICl^27^ and H_3_N···ICl.^28^ All have *C*_3v_ symmetry, with all atoms but the three H atoms of PH_3_ or NH_3_ lying on the *C* *a*3 axis and therefore all are isomorphic with H_3_P···AgI. The hydrogen-bonded analogues H_3_P···HI and H_3_N···HI have also been discussed in a detailed review,^56^ where it is concluded, based on several indirect observations, that there is little evidence of significant charge rearrangement or HI bond lengthening in these two complexes. Both are weakly bound, having quadratic force constants *F*_22_ = *F*_P···H_ or *F*_N···H_ of 3.4 N m^–1^ and 7.2 N m^–1^, respectively. These values are more than an order of magnitude smaller than those of H_3_P···AgI and H_3_N···CuI when *F*_P···Ag_ or *F*_N···Cu_ are calculated from the centrifugal distortion constant *D*0*J* by means of eqn (3), the more accurate method for strongly bound complexes. The related halogen-bonded H_3_P···ICl^27^ and H_3_N···ICl^28^ have *F*_22_ = *F*_P···I_ = 20.8 N m^–1^ and *F*_22_ = *F*_P···I_ 30.4 N m^–1^, respectively, when obtained by means of eqn (4). As indicated earlier, the larger is *F*_22_ relative to *F*_11_, the more serious will be its underestimation when eqn (4) is used. This underestimation is likely to be negligible for H_3_P···HI and H_3_N···HI, but it is possible that the values of *F*_22_ for H_3_P···ICl and H_3_N···ICl will both be somewhat larger (but only by a few %) than those reported previously. Clearly, the halogen-bonded complexes H_3_P···ICl and H_3_N···ICl are significantly more strongly bound than the hydrogen-bonded species H_3_P···HI and H_3_N···HI (when using the *F*_22_ criterion) but less so than H_3_P···AgI and H_3_N···CuI. According to a method of estimating electric charge redistribution from the changes in the I and Cl nuclear quadrupole coupling constants,^27,28,57^ there is a net movement of 0.15*e* (where *e* = electronic charge) from Cl to I in ICl when each of H_3_P···ICl and H_3_N···ICl is formed, thereby suggesting similar charge movement to that observed in each of H_3_P···AgI and H_3_N···CuI.
